# Association of the hsCRP- triglyceride-glucose index (CTI) with early infarct growth rate in acute ischemic stroke due to large artery occlusion

**DOI:** 10.3389/fnagi.2026.1917540

**Published:** 2026-08-26

**Authors:** Haiping Tang, Guojun He, Lei Li, Shuangfei You, Deming Li, Songjie Chen, Yan Guo, Yongqing Cheng, Shifu Sun

**Affiliations:** 1 Department of Neurology, Binghai County People’s Hospital, Yancheng, Jiangsu, China; 2 Department of Neurology, The Yancheng Clinical College of Xuzhou Medical University, The First People’s Hospital of Yancheng, Yancheng, Jiangsu, China

**Keywords:** acute ischemic stroke, biomarker, hsCRP-triglyceride-glucose index, infarct growth rate, large artery occlusion

## Abstract

**Background:**

Rapid infarct growth in acute ischemic stroke (AIS) due to large artery occlusion (LAO) is associated with poor clinical outcomes despite successful recanalization. The hypersensitive C-reactive protein (hsCRP)-triglyceride-glucose (TyG) index (CTI), a novel biomarker integrating insulin resistance and systemic inflammation, may reflect a meta-inflammatory state that exacerbates ischemic injury. However, its relationship with early infarct growth rate (IGR) remains unexplored. This study aimed to investigate whether CTI is independently associated with early IGR in patients with anterior circulation LAO.

**Methods:**

We conducted a prospective cross-sectional study of 247 patients with AIS due to anterior circulation LAO. CTI was calculated as the product of the TyG index and high-sensitivity CRP from fasting blood samples. Early IGR was defined as the baseline infarct core volume (rCBF <30%) divided by the time from symptom onset to imaging. Patients were categorized into fast (≥10 mL/h) or slow (<10 mL/h) IGR groups. Multivariable logistic regression was used to assess the independent association between CTI and fast IGR.

**Results:**

The fast IGR group had significantly higher CTI levels (median 5.396 vs. 5.177, *p* = 0.002). Spearman’s correlation revealed a positive association between CTI and IGR (r = 0.348, *p* < 0.001). After adjusting for confounders, CTI remained an independent predictor of fast IGR (adjusted OR = 1.635, 95% CI: 1.072–2.493, *p* = 0.022). Subgroup analyses confirmed the consistency of this association. ROC analysis showed an AUC of 0.613 (95% CI: 0.543–0.683) for CTI in discriminating fast IGR.

**Conclusion:**

Elevated CTI is independently associated with faster early infarct growth in LAO stroke, suggesting its potential role as an adjunctive biomarker that may complement clinical and imaging assessment for risk stratification. However, the modest discriminative performance (AUC = 0.613) underscores the need for integration with established predictors rather than standalone use.

## 1 Introduction

Ischemic stroke represents a major global health burden, characterized by high incidence, disability, and mortality rates (Islam et al., 2023). Among its various subtypes, acute ischemic stroke (AIS) due to large artery occlusion (LAO) is one of the most devastating conditions (Hilkens et al., 2024). LAO typically leads to a large territory of hypoperfusion, resulting in rapid progression of cerebral infarction and severe neurological deficits. The advent of endovascular thrombectomy (EVT) has significantly improved clinical outcomes for eligible patients with acute ischemic stroke (Nguyen et al., 2024). However, a substantial proportion of these individuals continue to experience poor functional recovery despite successful recanalization (Widimsky et al., 2023). This is often attributable to the rapid expansion of the core infarction before or after treatment, highlighting the critical importance of the early infarct growth rate (IGR) as a key determinant of final tissue fate and clinical prognosis (Pensato et al., 2025; Malikova et al., 2023a). Identifying available biomarkers capable of predicting rapid IGR at the initial stage is therefore crucial for risk stratification, optimizing therapeutic strategies, and potentially guiding novel neuroprotective interventions.

The dynamics of infarct progression are influenced by a complex interplay of factors, including collateral circulation status, blood pressure, and metabolic state (Pensato et al., 2025; Wouters et al., 2024). Among these, insulin resistance (IR) has emerged as a significant contributor to both stroke risk and pathogenesis (Ago et al., 2018). IR, a state of reduced physiological response to insulin, promotes a pro-inflammatory and pro-thrombotic milieu, exacerbating ischemic injury through mechanisms such as enhanced oxidative stress and endothelial dysfunction (Abdul-Ghani et al., 2024). The gold-standard method for assessing IR, the hyperinsulinemic-euglycemic clamp, is impractical for routine clinical use. The triglyceride-glucose (TyG) index, calculated from fasting triglyceride and glucose levels, has been validated as a simple, reliable, and cost-effective surrogate marker for IR (Bastard et al., 2012). Elevated TyG index has been associated with an increased risk of stroke incidence and poor functional outcomes (Wang et al., 2022; Yang et al., 2022). Nevertheless, its specific role in predicting the acute, dynamic process of infarct growth in the high-stakes context of LAO remains inadequately explored.

Concurrently, inflammation is a fundamental pathophysiological mechanism in AIS (Delong et al., 2022). C-reactive protein (CRP), a classic systemic inflammatory marker, rises rapidly after stroke onset. Elevated admission CRP levels have been consistently correlated with larger infarct volume, greater stroke severity, and worse long-term outcomes (Di Napoli et al., 2005; Bos et al., 2006). Importantly, IR and inflammation are not independent pathways; they interact closely, creating a synergistic “metabolism-inflammatory” state that can accelerate tissue damage (Bloomgarden, 2003). This interplay suggests that a composite index integrating markers of both metabolic dysfunction and inflammation might provide a more comprehensive prognostic tool than either marker alone.

Recently, the CRP-TyG index (CTI), a novel biomarker combining TyG index and CRP, has been proposed to reflect this meta-inflammatory state (Ruan et al., 2022). It has demonstrated superior predictive value for cardiovascular events and cancer compared to its individual components (Gao et al., 2025; Ruan et al., 2024). Recent studies have provided preliminary evidence linking the TyG index and CTI to stroke-related outcomes. Nam et al. demonstrated that a higher TyG index was independently associated with early recurrent ischemic lesions in AIS patients (Nam et al., 2021). More recently, Yang and Liu reported that higher cumulative CTI exposure was significantly associated with an increased risk of incident stroke (HR = 1.90), with persistently elevated CTI levels conferring an even higher risk (HR = 1.75) (Yang et al., 2022). However, the utility of the CTI in AIS, particularly its association with the rate of early infarction progression in LAO patients, is currently unknown.

Therefore, we conducted a prospective cross-sectional study to test the hypothesis that a higher CRP-TyG index (CTI), calculated from fasting blood drawn on the first morning after admission, is independently associated with a faster early infarct growth rate (IGR) in patients with anterior circulation LAO-induced AIS. If confirmed, CTI could serve as a readily available adjunctive biomarker for early identification of patients at high risk for rapid infarct progression, potentially enabling more personalized and timely therapeutic strategies when integrated with clinical and imaging assessment.

## 2 Material and methods

This single-center, prospective cross-sectional study was conducted in accordance with the principles of the Helsinki Declaration. It has been approved by the Ethics Committee of the First People’s Hospital of Yancheng, and all patients have given their informed consent.

### 2.1 Participants

**FIGURE 1:**
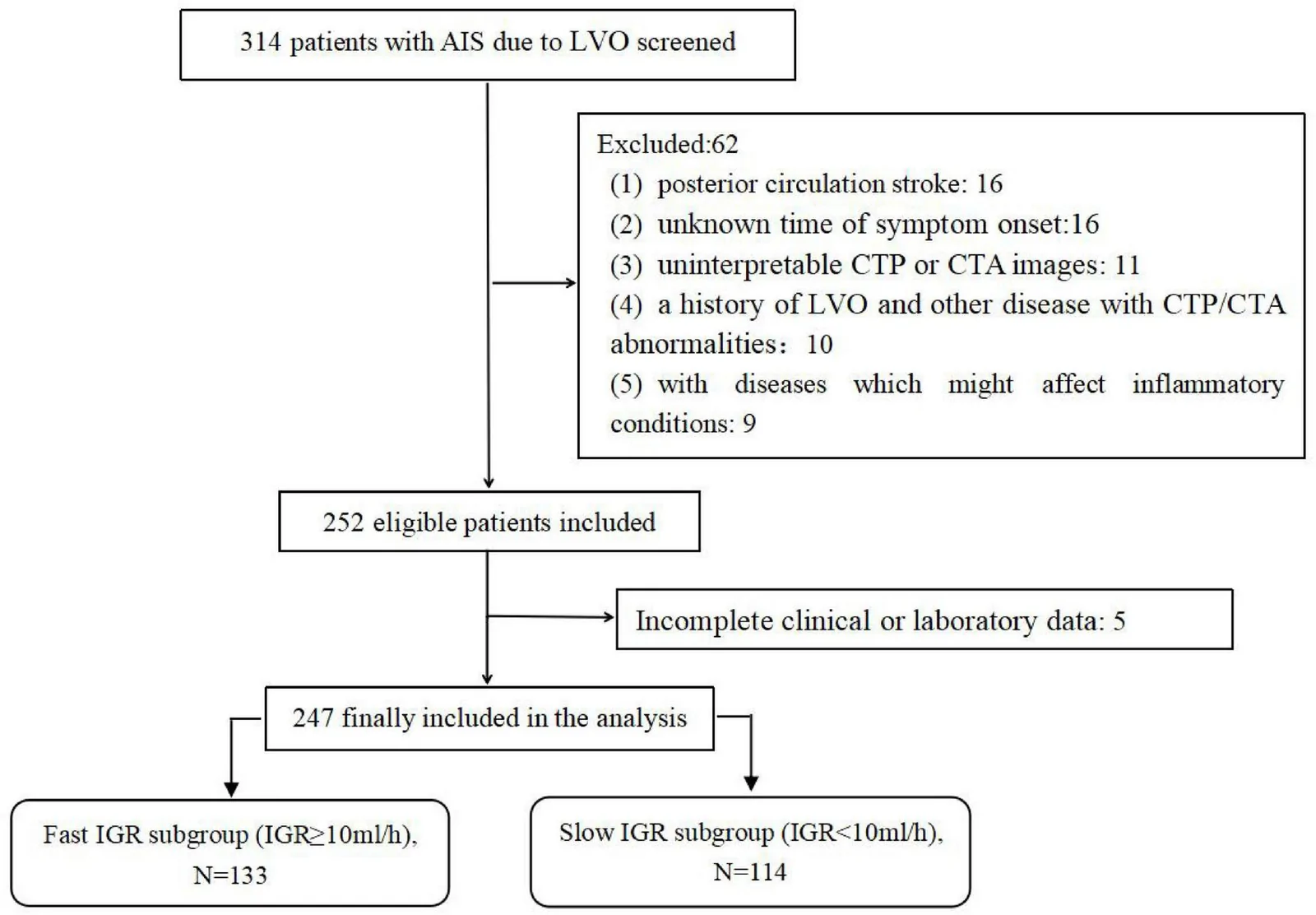
Flow chart of patient recruitment.

This study consecutively enrolled patients with acute ischemic stroke with large vessel occlusion (LVO) in the First People’s Hospital of Yancheng from October 2023 to November 2024. Figure 1 illustrates the enrollment flowchart of the participants in this study. The Inclusion criteria included: (1) age ≥18 years, (2) capable of cooperating to complete emergency CTP and CTA examinations, (3) a precise time of symptom onset within 8 h prior to the CTP/CTA scan, (4) occlusion of the intracranial internal carotid artery (ICA) or the M1/M2 segment of the middle cerebral artery (MCA), confirmed by computed tomography angiography (CTA). The exclusion criteria were as follows: (1) posterior circulation stroke, (2) unknown time of symptom onset, (3) significant motion artifacts on baseline CTP or CTA rendering images uninterpretable, (4) a history of LVO cerebral infarction, extensive cerebral hemorrhage, severe brain trauma, and other brain diseases that might affect the CTP assessment, (5) incomplete clinical or laboratory data required for analysis, (6) with diseases which might affect inflammatory conditions, such as acute infection, tumors, severe liver or kidney disease and autoimmune diseases.

### 2.2 Baseline data collection and clinical assessment

All baseline characteristics, including demographics (age, gender), relevant medical history [e.g., hypertension (HP), diabetes mellitus (DM), hyperlipidemia (HLP), stroke/transient ischemic attack (TIA), atrial fibrillation (AF) and coronary heart disease (CHD)], pharmacological treatment situation (anticoagulation and antiplatelet therapy), as well as smoking and alcohol consumption histories, were systematically collected using a pre-designed, standardized questionnaire and case report form administered at the time of enrollment. Stroke severity on admission was assessed using the National Institutes of Health Stroke Scale (NIHSS) score.

Furthermore, key laboratory parameters including fasting blood glucose, triglycerides, and C-reactive protein (CRP) were measured from a venous blood sample obtained under fasting conditions on the second morning after admission. This timing was chosen to standardize the fasting condition required for accurate calculation of the TyG index, as both triglyceride and glucose levels are sensitive to prandial status. While we acknowledge that hsCRP may begin to rise in response to acute cerebral ischemia by this time, its relatively slow turnover (half-life of approximately 19 h) suggests that the measured CTI may reflect a composite of both pre-existing chronic inflammation and early acute-phase response (Pepys and Hirschfield, 2003). Two laboratory technicians who were blind to the clinical data processed all the blood samples of the subjects in the laboratory department of our hospital.

### 2.3 Imaging data collection and processing

All patients underwent a standardized multi-modal computed tomography (CT) protocol for acute stroke on admission, performed on a Siemens Somatom Force CT scanner. The protocol sequentially included non-contrast CT (NCCT) to exclude hemorrhage and assess early ischemic signs, followed by whole-brain CT perfusion (CTP) and CT angiography (CTA). For CTP/CTA, a 50 ml bolus of iodinated contrast agent was injected intravenously at a rate of 5 mL/s. Dynamic scanning was performed with the following parameters: 80 kVp, 150 mAs, coverage of 160 mm in the z-axis, with a total acquisition time of 60 s to ensure full coverage of the first-pass and recirculation phases.

The raw CTP source data were automatically transferred to and processed using the Syngo.Via software package. All processing was performed by two trained neurologists/radiologists who were blinded to the patients’ clinical and laboratory data. The software algorithm automatically performed motion correction, denoising, and voxel-based curve deconvolution to generate parametric maps, including cerebral blood flow (CBF), cerebral blood volume (CBV), and time-to-maximum (Tmax).

The ischemic core volume at baseline was automatically calculated by the software as the volume of brain tissue with a severe reduction in perfusion, defined as a relative CBF (rCBF) of < 30% compared to the contralateral, unaffected hemisphere. The hypoperfused tissue volume (penumbra) was defined as the tissue with a Tmax delay > 6.0 s. The mismatch ratio between the penumbra and the core was also automatically calculated. All volumetric results (in milliliters) and color-coded maps were systematically reviewed for technical adequacy and the absence of significant artifacts before being recorded in the study database.

### 2.4 Definitions

The CRP-triglyceride-glucose index (CTI) was calculated as the product of the TyG index and the C-reactive protein (hs-CRP) level, using the formula: CTI = TyG index × CRP (mg/L) (Ruan et al., 2022). The TyG index itself was derived from fasting laboratory measurements as Ln [fasting triglycerides (mg/dL) × fasting glucose (mg/dL)/2] (Bastard et al., 2012). The early infarct growth rate (IGR), which quantifies the velocity of ischemic core expansion prior to treatment, was calculated by dividing the baseline ischemic core volume (mL; defined on admission CTP as tissue with rCBF < 30%) by the time from symptom onset to CTP acquisition (h) (Pensato et al., 2025). For further descriptive comparisons, patients were dichotomized into groups of fast (IGR ≥ 10 mL/h) versus slow (IGR < 10 mL/h) infarct growth (Pensato et al., 2025; Sarraj et al., 2021). The choice of 10 mL/h as the threshold was based on previously published literature demonstrating that this cutoff provides optimal discrimination for clinical outcomes, including malignant edema, hemorrhagic transformation, and poor functional recovery following endovascular thrombectomy (Sarraj et al., 2021; Pensato et al., 2025). Biologically, this threshold approximates the rate at which the ischemic penumbra may become irreversibly injured before therapeutic intervention can be implemented. Furthermore, the hypoperfusion intensity ratio (HIR) was assessed as a surrogate marker of collateral circulation status, calculated from the CTP data as the ratio of the volume of tissue with a Tmax delay > 10 s to the volume of tissue with a Tmax delay > 6 s (HIR = Tmax > 10 s volume/Tmax > 6 s volume) (Pensato et al., 2025).

### 2.5 Statistics

Continuous variables were presented as mean ± standard deviation or median with interquartile range (IQR) based on their normality distribution, which was assessed using the Shapiro–Wilk test. Categorical variables were summarized as frequencies and percentages. The primary analysis aimed to evaluate the correlation between the continuous CRP-triglyceride-glucose index (CTI) and the early infarct growth rate (IGR). Given the anticipated non-normal distribution of these variables, this association was quantified using Spearman’s rank correlation coefficient. Inter-group differences in baseline characteristics were compared using the Student’s *t*-test, Mann–Whitney U test, Chi-square test, or Fisher’s exact test, as appropriate.

Then, binary logistic regression models were employed to determine the independent association between CTI and fast infarct growth. First, univariate logistic regression was conducted to screen for potential risk factors associated with fast IGR. Variables with a significance level of *P* < 0.1 in the univariate analysis, along with the pre-specified clinically relevant confounders of age and sex, were entered into a subsequent multivariate logistic regression model. Multicollinearity diagnostics were performed by calculating variance inflation factors (VIFs) for all variables in the final multivariate model, with VIF > 5 indicating significant collinearity. Core infarct volume and time from last-known well to imaging were excluded from the multivariate model a priori due to inherent mathematical coupling with IGR, as IGR is directly calculated from these two parameters. Fasting TG and CRP were also excluded to avoid collinearity with CTI, which is a composite of these components. The results were expressed as odds ratios (OR) with their corresponding 95% confidence intervals (CI).

To evaluate the incremental predictive value of CTI beyond established clinical and imaging predictors, we performed Net Reclassification Improvement (NRI) and Integrated Discrimination Improvement (IDI) analyses. A baseline model was constructed including age, sex, atrial fibrillation, hypertension, previous stroke/TIA, ASPECTS, HIR, and NIHSS. An extended model was then built by adding CTI to the baseline model. The continuous NRI and IDI with 95% confidence intervals and *P* values were calculated using the PredictABEL package in R (version 4.2.0). To further validate the robustness of the association, we performed multivariable linear regression with IGR treated as a continuous variable, adjusting for the same covariates as in the logistic model. The results were expressed as beta coefficients (β) with 95% confidence intervals.

To verify the robustness of the primary findings, subgroup analyses were performed by stratifying patients based on age, sex, and key vascular risk factors. The consistency of the association between CTI and fast IGR across these subgroups was evaluated. Finally, the discriminatory capability of CTI for identifying fast infarct growth was assessed using receiver operating characteristic (ROC) curve analysis, and the area under the curve (AUC) was reported. A two-sided *p*-value < 0.05 was considered statistically significant. All statistical analyses were performed using SPSS version 26.0 and R version 4.2.0.

## 3 Results

### 3.1 Patient characteristics stratified by infarct growth rate

A total of 314 consecutive patients with acute ischemic stroke due to anterior circulation large vessel occlusion were prospectively enrolled. After applying the exclusion criteria, 67 patients were excluded, resulting in a final cohort of 247 subjects for analysis. The overall cohort had a median age of 72 years (IQR: 63–77), with 135 (54.7%) being male. The median CRP-TyG index (CTI) was 5.28 (IQR: 4.67–5.58), and the median infarct growth rate (IGR) was 10.92 mL/h (IQR: 5.95–17.04). Using the median IGR as a cutoff, 114 patients were categorized into the slow IGR group (<10 mL/h) and 133 into the fast IGR group (≥10 mL/h) (Figure 1).

Comparative analysis of baseline characteristics is detailed in Table 1. Patients in the fast IGR group were significantly older and had a higher prevalence of hypertension, atrial fibrillation, and previous stroke/TIA. Regarding stroke severity and imaging markers, the fast IGR group presented with significantly larger baseline core infarct volumes, shorter time from last-known well to imaging, lower ASPECTS on CTA, and higher hypoperfusion intensity ratio (HIR). Laboratory profiles further revealed that the fast IGR group had significantly elevated levels of triglycerides (TG), C-reactive protein (CRP), TyG index, and the composite CTI.

**TABLE 1:** Comparison of demographic and clinical characteristics between slow and fast progressors/IGR.

Baseline characteristics	Slow IGR (<10 ml/h) (*n* = 114)	Fast IGR (≥10 ml/h) (*n* = 133)	*P-value*
Demographics			
Male (%)	57 (50.0)	78 (58.6)	0.174
Age, years	69.0 (60.0, 77.0)	72.0 (66.0, 77.0)	0.048
Medical history (%)			
Hypertension	54 (47.4)	82 (61.7)	0.024
Diabetes mellitus	34 (29.8)	33 (24.8)	0.377
Hyperlipidemia	29 (25.4)	30 (22.6)	0.596
Coronary heart disease	6 (5.3)	15 (11.3)	0.091
Atrial fibrillation	34 (29.8)	61 (45.9)	0.010
Previous stroke/TIA	17 (14.9)	34 (25.6)	0.039
Smoking, (%)	30 (26.3)	30 (22.6)	0.732
Drinking, (%)	15 (13.2)	27 (20.3)	0.136
Medication use history, n (%)			
Antiplatelet drugs	40 (35.1)	56 (42.1)	0.259
Anticoagulant drugs	34 (29.8)	34 (25.6)	0.455
Clinical characteristics			
NIHSS on admission	15.0 (10.0, 20.25)	16.0 (11.0, 23.0)	0.189
SBP, (mmHg)	150.0 (136.0, 165.25)	147.0 (135.0,166.0)	0.319
DBP, (mmHg)	88.0 (79.0, 98.0)	86.0 (77.0, 96.0)	0.410
ICA occlusion, *n* (%)	42 (36.8)	53 (39.8)	0.628
Infarction core volume, median (rCBF < 30%) (IQR) (cm^3^)	25.765 (15.753, 37.460)	46.78 (35.475, 65.76)	<0.001
Time from last-known well to CT and CTP acquisition, median (IQR) (hour)	5.0 (3.3, 7.0)	3.0 (1.67, 4.5)	<0.001
ASPECTS on CTA, median (IQR)	3 (2, 4)	3 (2, 4)	0.003
HIR, median (IQR)	0.294 (0.178, 0.437)	0.440 (0.275, 0.574)	<0.001
HIR ≥ 0.4, *n* (%)	32 (31.6)	74 (55.6)	<0.001
Intravenous thrombolysis, n (%)	41 (36.0)	53 (39.8)	0.531
Endovascular thrombectomy, n (%)	54 (47.4)	59 (44.4)	0.591
Laboratory characteristics			
FPG, median (IQR) (mg/dl)	137.33 (128.750, 148.343)	137.34 (129.960, 145.085)	0.511
TG, median (IQR) (mg/dL)	92.483 (74.561, 145.669)	123.015 (86.730, 196.107)	0.001
CRP, median (IQR) (mg/dL)	1.824 (0.577, 4.992)	2.906 (0.963, 7.649)	0.031
TyG, median (IQR)	4.744 (4.592, 4.968)	4.859 (4.694, 5.116)	0.004
CTI, median (IQR)	5.177 (4.602, 5.472)	5.396 (4.765, 5.765)	0.002

IGR, infarct growing rate; TIA, transient ischemic attack; NIHSS, National Institutes of Health Stroke Scale; SBP, systolic blood pressure; DBP, diastolic blood pressure; ICA: internal carotid artery; CBF, cerebral blood flow; IQR, interquartile range; CT, computed tomography; CTP, computed tomography perfusion; ASPECTS, Alberta Stroke Program Early CT Score; CTA, computed tomography angiography; HIR, hypoperfusion intensity radio; FPG, fasting plasma glucose; TG, triglyceride; CRP, C-reactive protein; TyG, triglyceride glucose index; CTI, CRP-TyG index.

### 3.2 Association between CTI and IGR

**FIGURE 2:**
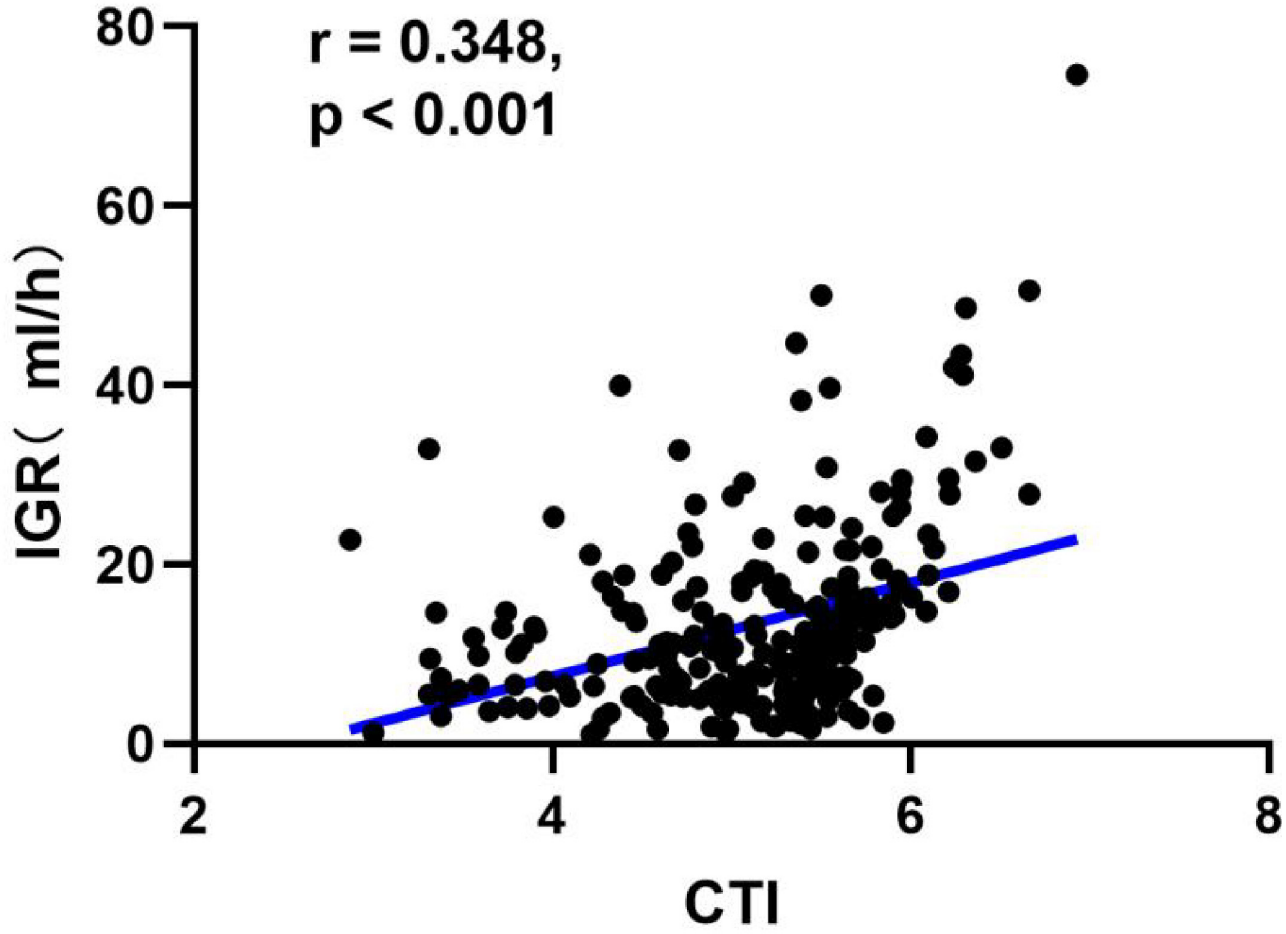
Spearman correlation analysis between CTI and IGR.

Spearman’s correlation analysis was subsequently performed to explore the correlation between CTI and IGR. The results revealed a statistically significant positive association between the CTI and IGR (*r* = 0.348, *p* < 0.001), as visualized in Figure 2.

### 3.3 Logistic regression analysis for fast IGR

Univariable logistic regression identified several variables associated with fast IGR, including age, hypertension, atrial fibrillation, previous stroke/TIA, ASPECTS on CTA, HIR, TG, CRP, TyG index, and CTI (Table 2). To avoid multicollinearity, TG, CRP, and the TyG index were excluded from the multivariable model alongside core infarct volume and time from last-known well to imaging.

**TABLE 2:** Univariate and multivariate logistic regression analyses for the potential risk factors associated with fast IGR including CTI as continuous variables.

Variable	Univariate analysis		Multivariate analysis	
	OR (95%CI)	*P* value	Adjusted OR (95%CI)	*P value*
Demographics				
Male	1.418 (0.857–2.347)	0.174	1,821 (1.014–3.272)	0.045
Age	1.025 (1.000–1.050)	0.050	1.012 (0.984–1.042)	0.405
Medical history				
Hypertension	1.786 (1.076–2.967)	0.025	1.345 (0.743–2.435)	0.328
Diabetes mellitus	0.776 (0.443–1.362)	0.378		
Hyperlipidemia	0.854 (0.475–1.533)	0.597		
Coronary heart disease	2.288 (0.857–6.109)	0.099	1.452 (0.500–4.214)	0.493
Atrial fibrillation	1.993 (1.177–3.375)	0.010	2.065 (1.108–3.852)	0.023
Previous stroke/TIA	1.960 (1.027–3.739)	0.041	1.751 (0.843–3.637)	0.133
Smoking	0.816 (0.456–1.460)	0.493		
Drinking	1.681 (0.845–3.345)	0.139		
Medication use history				
Antiplatelet drugs	1.345 (0.803–2.254)	0.260		
Anticoagulant drugs	0.808 (0.462–1.414)	0.455		
Clinical characteristics				
NIHSS on admission	1.028 (0.996–1.061)	0.086	0.994 (0.957–1.033)	0.766
SBP	0.993 (0.981–1.004)	0.208		
DBP	0.992 (0.973–1.011)	0.392		
ICA occlusion	1.136 (0.679–1.901)	0.628		
Infarction core volume, median (rCBF < 30%)	1.073 (1.053–1.095)	<0.001	–^a^	–^a^
Time from last-known well to CT and CTP acquisition	0.667 (0.583–0.762)	<0.001	–^a^	–^a^
ASPECTS on CTA	0.750 (0.615–0.914)	0.004	1.867 (1.112–3.136)	0.018
HIR*10	1.391 (1.206–1.604)	<0.001	2.100 (1.466–3.009)	< 0.001
Laboratory characteristics				
FPG	0.997 (0.990–1.004)	0.469		
Fasting TG	1.005 (1.002–1.008)	0.003	–^a^	–^a^
CRP	1.082 (1.017–1.151)	0.012	–^a^	–^a^
TyG	3.449 (1.449–8.208)	0.005	–^a^	–^a^
CTI	1.585 (1.113–2.256)	0.011	1.635 (1.072–2.493)	0.022

IGR, infarct growing rate; TIA, transient ischemic attack; NIHSS, National Institutes of Health Stroke Scale; SBP, systolic blood pressure; DBP, diastolic blood pressure; ICA: internal carotid artery; CBF, cerebral blood flow; IQR, interquartile range; CT, computed tomography; CTP, computed tomography perfusion; ASPECTS, Alberta Stroke Program Early CT Score; CTA, computed tomography angiography; HIR, hypoperfusion intensity radio; FPG, fasting plasma glucose; TG, triglyceride; CRP, C-reactive protein; TyG, triglyceride glucose index; CTI, CRP-TyG index; OR, odds ratio.

^a^Infarction core volume, time from last-known well to CT and CTP acquisition, fasting TG and CRP were excluded because of multicollinearity.

In the multivariable model (Table 2), which was adjusted for sex, age, and all variables with a *p* < 0.1 in univariable analysis, CTI remained an independent factor associated with fast IGR (adjusted OR = 1.635, 95% CI = 1.072–2.493, *p* = 0.022). Additionally, male sex, atrial fibrillation, and a higher HIR were identified as independent predictors, while a higher ASPECTS on CTA was associated with a reduced risk. The final model demonstrated acceptable collinearity, with all VIFs below 5 (CTI: 1.114, ASPECTS: 3.768, HIR: 3.858, age: 1.180, sex: 1.047, atrial fibrillation: 1.140, hypertension: 1.124, previous stroke/TIA: 1.054, NIHSS: 1.171, coronary heart disease: 1.044), indicating that multicollinearity did not substantially affect the estimates.

### 3.4 Sensitivity analysis: IGR as a continuous variable

To further validate the robustness of the association between CTI and infarct growth, we performed multivariable linear regression with IGR treated as a continuous variable. After adjusting for age, sex, hypertension, atrial fibrillation, previous stroke/TIA, ASPECTS, HIR, and NIHSS, CTI remained significantly associated with IGR (β = 4.621, SE = 0.865, *p* < 0.001). The full model explained 22.1% of the variance in IGR (adjusted R^2^ = 0.191, F (9, 237) = 7.453, *p* < 0.001). Other independent predictors included HIR (β = 1.537, SE = 0.732, *p* = 0.037) and NIHSS (β = 0.169, SE = 0.080, *p* = 0.036). These results are consistent with the findings from our primary logistic regression analysis (Supplementary Table 1).

### 3.5 Subgroup analyses

**FIGURE 3:**
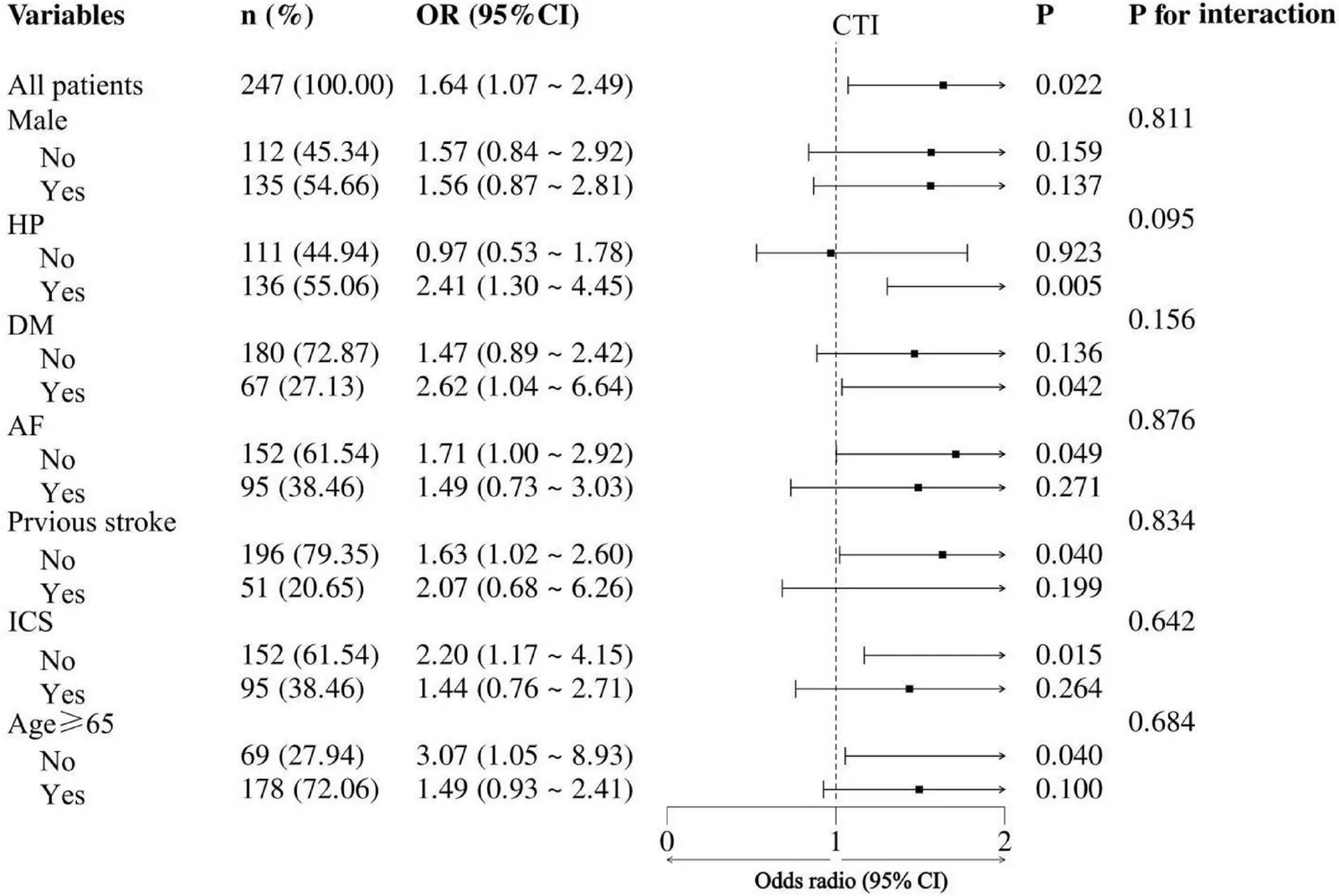
Subgroup analyses of the association between CTI and fast IGR among various patient groups.

Subgroup analyses were performed to assess the consistency of the association between CTI (as a continuous variable) and fast IGR across various patient demographics and clinical histories. As shown in Figure 3, no significant interaction effects were observed in subgroups stratified by sex, age, history of hypertension, diabetes, atrial fibrillation, stroke, or internal carotid artery occlusion status (all *p* for interaction > 0.05). This indicates that the positive association between CTI and fast IGR was robust and consistent across these different patient subgroups.

### 3.6 Discriminative and incremental predictive value of CTI

**FIGURE 4:**
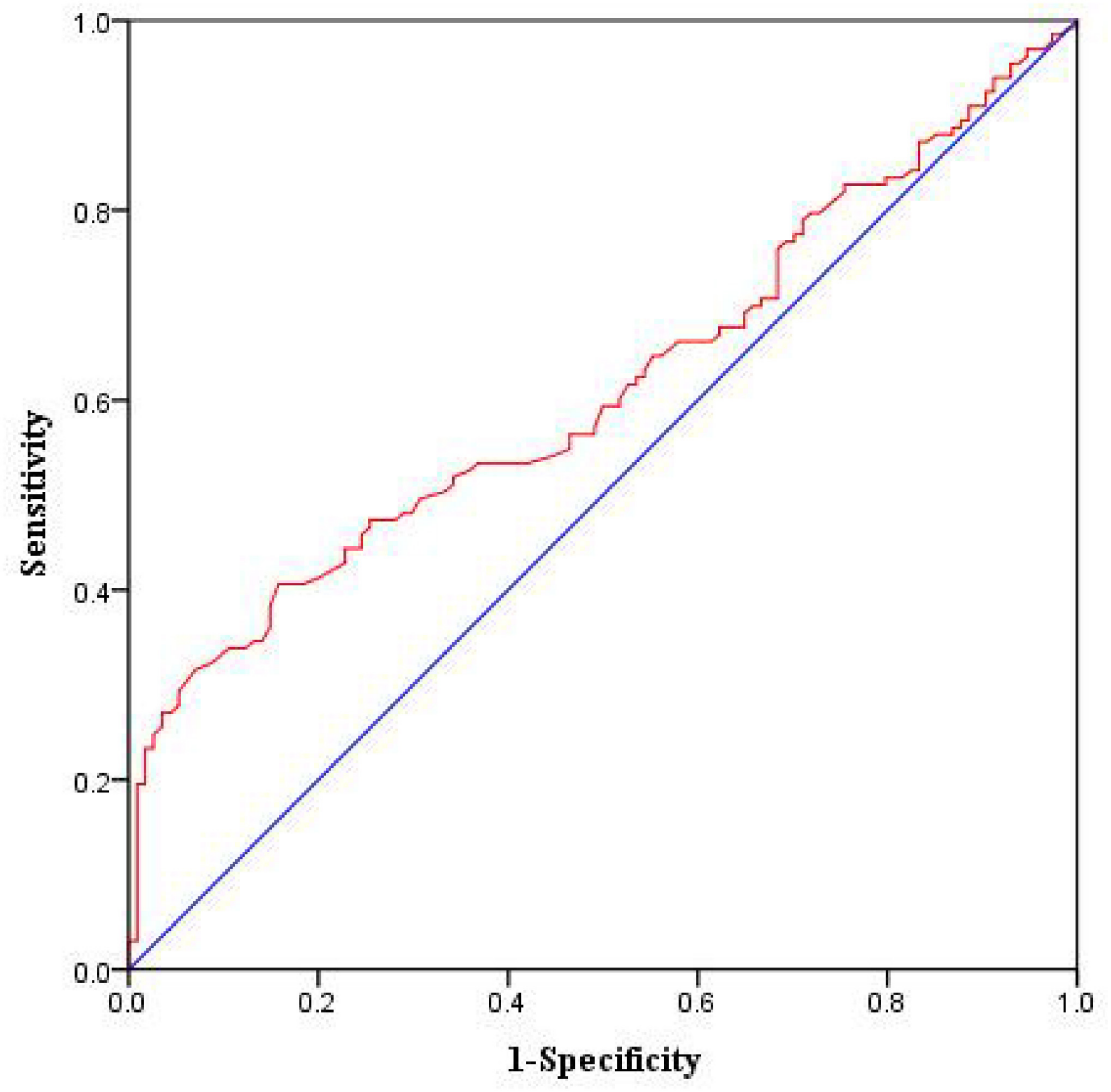
ROC analysis to evaluate the predictive value of CTI for fast IGR.

The ability of CTI to discriminate between slow and fast IGR phenotypes was evaluated using receiver operating characteristic (ROC) curve analysis. The area under the curve (AUC) was 0.613 (95% CI: 0.543–0.683, *p* = 0.036) (Figure 4). The optimal CTI cutoff value was determined to be ≥5.545, which provided a specificity of 84.2% and a sensitivity of 40.6%.

**FIGURE 5:**
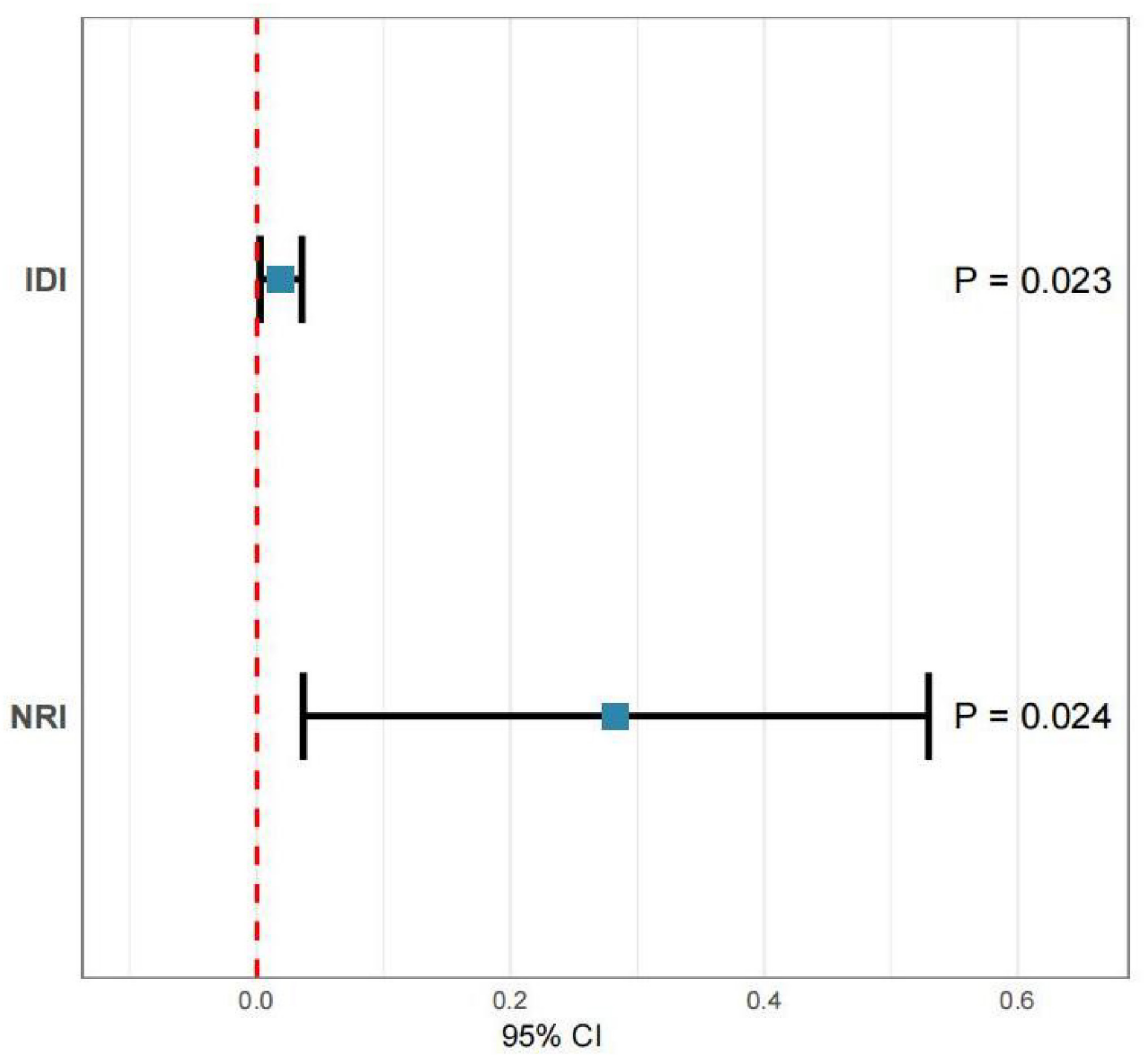
Forest plot of incremental predictive value of CTI.

To evaluate whether CTI provides incremental predictive value beyond established clinical and imaging predictors, we performed NRI and IDI analyses. A baseline model was constructed including age, sex, atrial fibrillation, hypertension, previous stroke/TIA, ASPECTS, HIR, and NIHSS. Adding CTI to this model yielded a continuous NRI of 0.283 (95% CI: 0.037–0.530, *p* = 0.024) and an IDI of 0.019 (95% CI: 0.003–0.036, *p* = 0.023), indicating that CTI provides a statistically significant improvement in risk reclassification and overall model discrimination beyond established predictors (Figure 5).

## 4 Discussion

This prospective cohort study provides novel evidence that the CRP-TyG index (CTI), an integrative biomarker of metabolic-inflammatory status, is independently associated with faster infarct growth in the hyperacute phase of anterior circulation large vessel occlusion stroke. Our analysis demonstrates several key findings in a logical sequence. First, we established a significant positive correlation between CTI levels and the infarct growth rate (IGR). Moreover, this association persisted as an independent risk factor even after comprehensive adjustment for established predictors, including ASPECTS and hypoperfusion intensity. Furthermore, the consistency of this relationship across all clinical subgroups underscores the robustness of our results. Finally, the CTI exhibited modest discriminatory performance and provided statistically significant incremental predictive value when added to established predictors, as demonstrated by NRI and IDI analyses. These findings collectively suggest that CTI may serve as a practical adjunctive biomarker for early pathophysiological stratification in LAO acute ischemic stroke, though its clinical utility should be considered in conjunction with established predictors rather than as a standalone tool.

In the context of large artery occlusion (LAO), IGR is not a uniform process but can be conceptually divided into early and late phases. The early or hyperacute IGR is typically defined as the rate of infarct expansion from symptom onset to the initial imaging assessment, often calculated as the baseline core infarct volume (e.g., on CT perfusion or diffusion-weighted imaging) divided by the time from onset to imaging (Pensato et al., 2025; Malikova et al., 2023a). In contrast, the late IGR refers to the progression occurring after the initial imaging, often assessed by comparing the final infarct volume on follow-up imaging (e.g., 24–72 h MRI) with the initial core volume (Rios Rocha et al., 2024).

Our study specifically focused on the hyperacute IGR, calculated from the baseline CTP core volume and the onset-to-imaging time. This choice is predicated on its unique pathophysiological significance. The hyperacute phase represents the crucial window where the fate of the ischemic penumbra is most actively determined, primarily by the adequacy of collateral circulation. A rapid hyperacute IGR signifies failure of compensatory mechanisms and reflects inherent tissue vulnerability before therapeutic intervention, independently predicting early neurological deterioration and poor outcomes despite successful recanalization (Pensato et al., 2025). Patients exhibiting fast hyperacute IGR face substantially higher risks of malignant edema, hemorrhagic transformation, and mortality (Huang et al., 2025; He et al., 2024; Sarraj et al., 2021). While previous investigations have identified various factors influencing IGR including hemodynamic parameters, metabolic factors, and collateral anatomical variations, many of these require advanced imaging quantification or represent transient physiological states, highlighting the need for readily available biomarkers that can integrate these complex pathophysiological processes (Rios Rocha et al., 2024; Pensato et al., 2025; Mohammaden et al., 2023).

The CTI emerges as an innovative solution to this clinical need, combining the TyG index, a well-validated surrogate of insulin resistance, with CPR, a cornerstone inflammatory biomarker. This composite design conceptually stems from the recognized synergy between metabolic dysfunction and chronic inflammation, termed “metabolism-inflammation.” The principal advantage of the CTI over its individual components lies in its ability to capture this pathophysiological synergy, potentially offering a more holistic reflection of the underlying deleterious state than either marker alone (Ruan et al., 2022; Zhao, 2023). Currently, the application of CTI in clinical practice remains in the investigational phase, with the most robust evidence derived from research on cardiovascular diseases and oncology (Ruan et al., 2022; Zhao, 2023; Gao et al., 2025). A few studies have shown that elevated CTI may increase the risk of stroke in specific populations (Huo et al., 2025; Tang et al., 2024). The prognostic value of the TyG index and CTI is supported by recent evidence. Nam et al. found that a higher TyG index was independently associated with early recurrent ischemic lesions (aOR 2.63), suggesting that insulin resistance promotes recurrent ischemic injury through atherosclerosis-related mechanisms (Nam et al., 2021). At the population level, Yang and Liu demonstrated that higher cumulative CTI exposure was associated with a 90% increased risk of incident stroke, with a clear dose-response relationship (Yang and Liu, 2025). These findings align with our observation that elevated CTI at admission is associated with faster infarct progression, suggesting that CTI may capture both chronic vulnerability and acute exacerbation of the meta-inflammatory state. Meanwhile, its application in ischemic stroke remains largely unexplored. To our knowledge, no prior investigation has examined its relationship with hyperacute infarct growth dynamics in large artery occlusion stroke, making our study the first to establish this clinically relevant association.

The observed association between elevated CTI and accelerated infarct growth may be explained by several interconnected pathways that could amplify the ischemic cascade. We hypothesize that the IR component may induce endothelial dysfunction by reducing nitricoxide bioavailability and promoting pro-thrombotic states, potentially compromising collateral vasodilation and microvascular perfusion in the context of acute large vessel occlusion (Hill et al., 2021). Concurrently, the chronic inflammatory state primes for intensified post-occlusion inflammation, facilitating leukocyte infiltration that causes capillary plugging and further impairs penumbral perfusion (Bloomgarden, 2003). Critically, the potential meta-inflammatory synergy could create a vicious cycle that amplifies oxidative stress through mitochondrial dysfunction and reactive oxygen species generation (Furie and Inzucchi, 2008; Thomas et al., 2023). Therefore, patients with high CTI likely experience ischemia with pre-existing endothelial dysfunction, inflammatory priming, and elevated oxidative stress burden, collectively predisposing to rapid cellular collapse upon flow cessation. However, these mechanistic interpretations remain speculative and require validation through experimental and interventional studies.

The principal clinical implication of our findings lies in CTI’s practicality and accessibility. Derived from routine, inexpensive blood parameters available in most medical institution, it enables rapid risk stratification at admission. The high specificity at the optimal cutoff suggests particular utility in identifying patients with lower likelihood of rapid infarction progression, potentially guiding decisions about monitoring intensity and management strategies. While the modest sensitivity precludes its use as a standalone rule-in tool. The significant continuous NRI (0.283, *P* = 0.024) and IDI (0.019, *P* = 0.023) indicate that CTI adds statistically meaningful prognostic information beyond established clinical and imaging predictors, supporting its value as a complementary biomarker in multifactorial assessment. The modest effect sizes observed in these incremental analyses are consistent with the notion that infarct growth is a multifactorial process, and no single biomarker can fully capture its complexity. Furthermore, the consistent association observed across all clinical subgroups including those stratified by age, sex, comorbidities, and occlusion site, underscores the robustness and generalizability of our findings across heterogeneous stroke populations.

Our findings add to a growing body of literature investigating the utility of readily available metabolic and inflammatory biomarkers for risk stratification in ischemic stroke. For instance, the atherogenic index of plasma (AIP), calculated from routine lipid parameters, has recently been shown to predict mortality in ischemic stroke patients, highlighting the prognostic potential of inexpensive composite metabolic indices (Tatar et al., 2025). Similarly, studies have demonstrated that the TyG index, a component of CTI, is associated with stroke recurrence and poor functional outcomes (Wang et al., 2022; Yang et al., 2022). The CTI extends this paradigm by integrating both metabolic and inflammatory dimensions, potentially capturing the synergistic effects of these two pathological processes. Beyond systemic biomarkers, recent evidence has highlighted the importance of subclinical cardiac dysfunction in the pathophysiology of acute ischemic stroke. Advanced echocardiographic markers, such as diastolic global longitudinal strain (GLS), have been associated with stroke risk and may reflect early myocardial involvement that precedes overt cardiac disease (Ozkan et al., 2025). This observation underscores the intricate brain-heart connection and suggests that cardiac functional assessment may contribute to a more comprehensive risk stratification approach. While our study focused on a systemic metabolic-inflammatory biomarker (CTI), integrating such biomarkers with cardiac functional assessment could potentially provide a more holistic evaluation of stroke risk and prognosis. Future multimodal studies combining systemic biomarkers, advanced echocardiography, and neuroimaging may better elucidate the complex pathways linking cardiovascular health to cerebral ischemic injury.

Although our study provided epidemiological evidence for the correlation between CTI and IGR in patients with anterior circulation LAO ischemic stroke, there are still some limitations that need to be taken into account. Firstly, the single-center design necessitates external validation in larger, multi-center cohorts to confirm generalizability. As a cross-sectional analysis, our study demonstrates association rather than causation, and the temporal relationship between CTI and IGR cannot be definitively established. Secondly, Although we performed meticulous adjustment for recognized confounders, residual confounding from unmeasured factors cannot be entirely excluded. Thirdly, the timing of blood sampling-performed on the second morning after admission-was chosen primarily to standardize the fasting condition required for accurate calculation of the TyG index. However, we recognize that by this time, hsCRP may have begun to rise in response to acute cerebral ischemia, which introduces a potential confound in interpreting CTI as purely reflective of baseline metabolic-inflammatory status. While hsCRP has a relatively slow turnover and typically does not reach peak levels until 48–72 h after an acute event, we cannot exclude the possibility that the measured CTI partly reflects the early acute-phase response to infarction. Future investigations incorporating pre-stroke baseline inflammatory markers or serial post-stroke measurements would be valuable to clarify the temporal relationship between CTI and infarct growth dynamics. Fourth, although our primary analysis focused on pre-treatment predictors of early IGR, we acknowledge that post-treatment factors-including intravenous thrombolysis, endovascular thrombectomy, successful reperfusion (mTICI grade), and procedural times, may also influence infarct evolution and clinical outcomes. While these variables are not directly relevant to the prediction of pre-treatment IGR, they represent important modifiers of the overall disease trajectory. Additionally, while the IGR calculation based on CT perfusion core volume and onset-to-imaging time represents a well-validated approach, it remains an estimation of the true biological growth rate. Future studies incorporating serial imaging assessments could provide deeper insights into the dynamic relationship between meta-inflammatory status and infarct evolution.

In conclusion, our prospective study establishes CTI as a novel, independent biomarker associated with hyperacute infarct growth rate in anterior circulation large vessel occlusion stroke. The CTI may reflect a pre-stroke metabolism-inflammatory state that could exacerbate tissue vulnerability through convergent pathways including endothelial dysfunction, impaired collateral perfusion, neuroinflammation, and oxidative stress, although this interpretation requires further investigation. As an inexpensive, readily available metric, CTI holds significant promise for early risk stratification, potentially guiding intensive monitoring and personalized management strategies aimed at mitigating one of the most aggressive forms of acute ischemic stroke. Future research should focus on validating these findings in diverse populations and exploring the potential for targeting meta-inflammatory pathways as a therapeutic strategy in acute stroke care.

## 5 Conclusion

In patients with acute ischemic stroke due to large artery occlusion, a higher CTI is associated with a faster early IGR. The CTI may reflect a pre-stroke metabolism-inflammatory state that could exacerbate tissue vulnerability. As an inexpensive, readily available metric, CTI holds promise as an adjunctive tool for early risk stratification, potentially guiding intensive monitoring and personalized management strategies when integrated with established clinical and imaging predictors.

## Data Availability

The raw data supporting the conclusions of this article will be made available by the authors, without undue reservation.
