# Supplementary material for: Association of the hsCRP- triglyceride-glucose index (CTI) with early infarct growth rate in acute ischemic stroke due to large artery occlusion
Source: Front Aging Neurosci. 2026 Aug 26;18:1917540. doi: 10.3389/fnagi.2026.1917540 (PMC13553777; doi:10.3389/fnagi.2026.1917540)
Supplement: Supplementary file 1 [file Data_Sheet_1.pdf]

Supplementary Material

1 Supplementary Tables

Supplementary Table S1. Multivariable linear regression analysis for factors associated with IGR (continuous)

Variable	β	SE	t	*P*
CTI	4.621	0.865	5.339	<0.001
Age	-0.004	0.062	-0.065	0.948
Gender	1.732	1.238	1.399	0.163
Hypertension	1.566	1.280	1.223	0.223
Atrial fibrillation	2.462	1.319	1.866	0.063
Previous stroke/TIA	0.957	1.527	0.627	0.531
ASPECTS	1.187	1.089	1.090	0.277
HIR (per 0.1 increase)*	1.537	0.732	2.100	0.037
NIHSS	0.169	0.080	2.112	0.036

Notes: *: HIR was scaled by a factor of 10 for model fitting, coefficient reflects per 0.1 unit increase.
Abbreviations: IGR, infarct growing rate; TIA, transient ischemic attack; NIHSS, National Institutes of Health Stroke Scale; ASPECTS, Alberta Stroke Program Early CT Score; HIR, hypoperfusion intensity radio; CTI, C-reactive protein (hsCRP)-triglyceride-glucose (TyG) index; SE, standard error.
